# IL-4Rα deletion disrupts psychomotor performance and reference memory in mice while sparing behavioural phenotype associated with spatial learning

**DOI:** 10.1016/j.bbi.2020.12.003

**Published:** 2021-02

**Authors:** T.M. Brombacher, D.C. Ajonijebu, M. Scibiorek, I. Berkiks, B.O. Moses, T. Mpotje, F. Brombacher

**Affiliations:** a International Centre for Genetic Engineering and Biotechnology (ICGEB), Cape Town Component, South Africa; b Division of Immunology, Institute of Infectious Disease and Molecular Medicine (IDM), Health Science Faculty, University of Cape Town, 7925, South Africa

**Keywords:** IL-4Rα, Interleukin-4 receptor alpha, IL-13, interleukin-13, IFN-γ, interferon gamma, BDNF, brain-derived neurotrophic factor, MWM, Morris water maze, STAT4, signal transducer and activator of transcription 4, GABAergic, γ-aminobutyric-acid, mRNA, messenger ribonucleic acids, Interleukin-4 receptor alpha, Interleukin-13, Interferon gamma, Brain-derived neurotrophic factor, Myeloid cells, Macrophages, Microglia, Spatial learning, Acquisition, Reference memory, Cognition, Cytokines, Morris water maze

## Abstract

Contribution of immune mediators, interleukin-4 and interferon gamma to cognitive functioning is receiving increasing attention. However, the fundamental question about how heterodimeric interleukin-4 receptor alpha– and interferon gamma– producing myeloid cells converge to influence hippocampal–dependent spatial memory tasks through immunomodulation of multisensory inputs from other brain areas remains unexplored. Here, we show that mice lacking interleukin-4 receptor alpha are able to successfully learn spatial tasks, while reference memory is impaired. Moreover, the absence of interleukin-4 receptor alpha leads to simultaneous increase in proportions of CD11b + myeloid cells in the hippocampus and thalamus, but not the brainstem during acquisition. Interleukin-4 receptor alpha deletion significantly decreased expression of myeloid cell–derived interferon gamma in the thalamus during the acquisition phase and simultaneously increased brain-derived neurotrophic factor production in the thalamus and brainstem of trained mice. We provide evidence that interleukin-4 receptor alpha is essential for cognitive performance while training–induced alterations in interferon gamma activity and brain-derived neurotrophic factor signalling may contribute to neuromodulation of learned tasks and consequently affect systems–level memory encoding and consolidation.

## 1 Introduction

Multiple lines of inquiry have demonstrated that certain components of the immune system contribute significantly to homeostatic regulation of brain functions. Of importance are interleukin (IL)-4 and interferon gamma (IFN-γ) cytokines recently identified as key players in neuromodulation of cognitive behaviours involving spatial learning tasks and memory (Gadani et al., 2012).

IL-4 is a pleiotropic polypeptide actively involved in regulating responses of lymphocytes, myeloid cells and non-hemopoietic cells (Junttila, 2018). Besides the long-known roles of IL-4 in the management of inflammatory diseases, its pro-cognitive roles in the brain are well documented (Derecki et al., 2011, 2010; Li et al., 2017). Evidence from a previous animal study indicates that mice lacking IL-4 exhibited skewed proinflammatory meningeal myeloid cell phenotype and cognitive impairment in visuo-spatial learning tasks, which was reversed by transplantation of IL-4–competent bone marrow (Derecki et al., 2010). Recent findings from our laboratory further confirmed that immune–driven cognitive functions are not limited to IL-4 producing T cells, but rather complex immunological signalling cascade events essentially involve IL-13 recruitment for optimal acquisition of learned tasks (Brombacher et al., 2017). Biological effects of these two sister cytokines (IL-4 and IL-13) are mediated by shared signalling via the common heterodimeric interleukin-4 receptor (IL-4R) α-chain (Brombacher et al., 2017, Gadani et al., 2012). Interestingly, more recent studies have associated IL-4Rα signalling with cytoskeletal remodelling and axonal repair in neuroinflammatory disease model, and its beneficial impact on brain homeostasis and behaviour (Vogelaar et al., 2018, Zipp et al., 2019). Despite this, little is known about the specific roles of IL-4Rα in cognitive neuroscience and merits further investigation.

IFN-γ is a prototypical macrophage-activating cytokine that regulates variety of important immunological programs (Munder et al., 1998). Under certain conditions, IFN-γ messenger ribonucleic acids

(mRNA) and protein have been detected in various populations of mononuclear phagocytes or macrophages (Bogdan et al., 2020, Munder et al., 1998, Ohteki et al., 1999), including microglia which also express IFN-γ receptors (Filiano et al., 2016). One cellular mechanism of IFN-γ action in the brain is augmentation of tonic inhibitory currents. For instance, research findings by Filiano and colleagues (2016) reveal that IFN-γ–driven GABAergic (γ-aminobutyric-acid) currents constitute the molecular link between meningeal immunity and neural circuits influencing social behaviour (Filiano et al., 2016). A more recent study confirmed that disturbed neuronal excitability and neuroinflammation–induced cognitive dysfunction are associated with potentiation of GABAergic inhibitory signals in the hippocampal neurons by IFN-γ (Flood et al., 2019). This may account for the widely reported negative correlations between IFN-γ and cognition (Flood et al., 2019, Monteiro et al., 2016, Wilson et al., 2018), only with very limited contradictions (Litteljohn et al., 2014).

IL-4, one of the well-known inhibitors of macrophage function, has been shown to decrease IFN-γ production by mouse peritoneal macrophage through activation of signal transducer and activator of transcription 4 (STAT4) nuclear translocation (Bogdan et al., 2020). Similarly, behavioural defects of IFN-γ deficiency has been demonstrated to contrast with IL-4–mediated spatial learning tasks (Ivashkiv, 2018) which suggest a possible opposing action for these cytokine regulators. However, the fundamental mechanisms by which dysfunctional IL-4/IL-4Rα pathway may interfere with IFN-γ-producing microglia and/or myeloid cell functions and contributes to learning and memory remains unclear.

Since learning is essential for survival, some aspects of memory encoding and retrieval are not dependent entirely on hippocampal neurocircuitry and cortical networks but may potentially involve reorganization and modulation of multisensory inputs from other brain areas, particularly thalamus and brainstem. While recent demonstration indicated that optogenetic stimulation of GABAergic nucleus incertus neurons of the brainstem or their specific fibre projections in the hippocampus prevented formation of fear memories and altered memory encoding-related hippocampal rhythms (Szőnyi et al., 2019), Yagi et al (1998) previously showed that lesioning the brainstem magnocellular reticular formation significantly impaired avoidance learning in aged mice (Yagi et al., 1998). Other ablation studies reported that different functional areas of the thalamus provide sensory inputs that directly or indirectly contribute to acquisition of spatial memory (Lopez et al., 2009) or transformational processes leading to hippocampal-dependent memory engrams (Loureiro et al., 2012). To date, experimental data demonstrating the influence of brainstem/thalamic-myeloid cell-derived IFN-γ immunologic functions on hippocampal–dependent spatial memory and learning is lacking. This led us to theorize that training–induced alterations in IFN-γ activity by multisensory processing brain areas may contribute to immunomodulation of learned tasks and consequently affect systems-level memory encoding and consolidation. Based on this theoretical framework, we used loss-of-function approach to assess the largely unexplored role of IL-4Rα on psychomotor learning and spatial memory, with reference to endogenous brain derived neurotrophic factor (BDNF) production and neuromodulation of hippocampal–dependent engrams by brainstem–thalamic–myeloid cell–derived IFN-γ.

## 2 Materials and methods

### 2.1 Animals

Inbred 8-wk-old IL-4Rα–deficient (Barner et al., 1998), and wild-type control mice, of BALB/c genetic background, were obtained from the University of Cape Town specific pathogen-free animal facility, and kept in individually ventilated cages. All animals were housed in temperature- and humidity-controlled rooms, maintained on a 12 h light/dark cycle and age matched in each experiment. Animal protocols were approved by the independent Animal Ethics Research Committee at the University of Cape Town (approval no. 015/050), and all methods were performed in accordance with the relevant guidelines and regulations.

### 2.2 Morris water maze

Spatial learning and reference memory function of n = 6 mice per group were investigated in the MWM for eight days. During acquisition (learning), mice were given four 5-min trials a day for 4 days to locate a submerged plexiglass circular platform (10 cm in diameter), that was removed on day 5 to test for reference memory as previously detailed (Brombacher et al., 2017). Data were recorded using the EthoVision XT 8 automated tracking system (Noldus Information Technology, Leesburg, VA), and statistical analyses performed using Student *t* test , or ANOVA, with the Bonferroni post hoc test. Groups were run in alternating order on successive training days, with all MWM experiments conducted between 9:00 am and 3:00 pm during the lights-on phase. Shown are representative experiments out of a minimum of at least three independently performed in each case.

### 2.3 Brain sample collection

On either day 4 of day 5 post MWM spatial task, mice were euthanized with halothane to collect brain tissue samples following transcardial perfusions (Gage et al., 2012). Various brain areas were collected into CentriStar™ cap 15 ml Corning® centrifuge tubes (Corning, NY) in Isove’s Modified Dulbecco’s Medium (IMDM) (GIBCO/Invitrogen; Carlsbad, CA), 10% Fetal Calf Serum (FCS), and penicillin streptomycin (P/S) on ice. Samples from the brainstem, hypothalamus, and thalamus were collected according to modified established protocols (Gage et al.,2012, Rousseau and Caravagna, 2015). Tissue was pushed through 40 μm nylon cell strainers (Falcon®, Corning Incorporated, NY), and centrifuged at 1200 rpm at 4 °C for 10 min. Samples were re-suspended in 450 μl IMDM buffer for flow cytometry and snap frozen for enzyme-linked immunosorbent assay (ELISA) experimentation.

### 2.4 ELISA analysis

Single-cell suspensions from the meninges, brainstem, hypothalamus, and thalamus of MWM-trained and nontrained mice were analysed for BDNF levels according to the manufacturer’s instructions (Promega, Madison, WI). Plates were developed using a Versamax microplate spectrophotometer (Molecular Devices).

### 2.5 Flow cytometry

Flow cytometry was used to determine myeloid, macrophage, and microglia populations in single-cell preparations of the meninges, brainstem, hypothalamus, and thalamus in complete media: Iscove Modified Dulbecco Media (Life Technologies/Invitrogen, Carlsbad, CA), 10% Fetal Calf Serum, penicillin/streptomycin on ice. Samples were stained with an Ab mix (MACS buffer plus 2% inactivated rat serum), 2% anti-FcγII/III (clone 2.4G2), anti-CD11b (clone M1/70; BD Horizon^TM^), anti-CD45 (clone 30-F11; BD Pharmingen), anti-IL-13 (clone eBio13A; eBioscience), and IFN-γ (clone MG1.2; BD Pharmingen) for 45 min on ice, and then fixed in 2% paraformaldehyde before being permeabilized (saponin containing permeabilization buffer) for 1 h, at 4 °C. Samples were read using a BD FACS Fortessa machine (BD Biosciences, San Diego, CA), and data analysed by FlowJo (Tree Star, Ashland, OR) to be graphed with GraphPad Prism software.

### 2.6 Statistical significance

GraphPad prism software (version 6.0) was used for statistical analyses. Shapiro-Wilk normality test was used to determine normal distribution of datasets. Data from behavioural experiments, FACS and ELISA were analysed using two-tailed unpaired Student’s *t-test*, Two-way analysis of variance (ANOVA) or repeated measures (RM) two-way ANOVA, corrected for multiple comparisons with a Bonferroni post-hoc, where applicable. Results were generated from three independent experiments and all data are reported as mean ± SEM, while *p <* 0.05 was considered statistically significant.

## 3 Results

### 3.1 IL-4Rα deletion impaired psychomotor performance and spatial reference memory

**Fig. 1:**
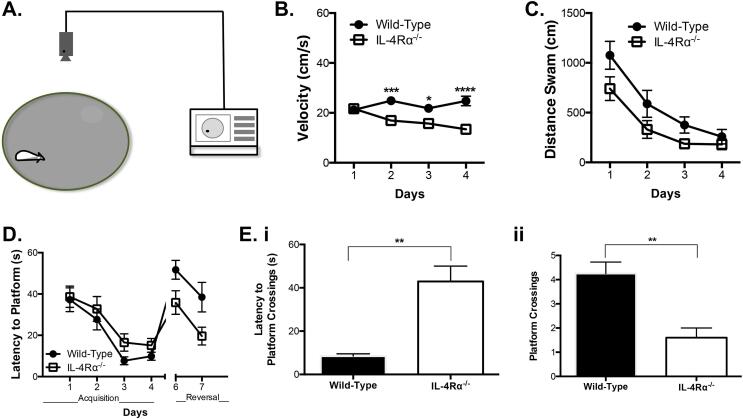
*Interleukin-4 receptor alpha induced cognitive defects.* Male mice (8 wks old) were investigated for learning and reference memory in the MWM. (a) A schematic diagram of the spatial task by MWM, with a water bath for mice to swim, and an over-viewing camera attached to a computer software. As a control factor to determine cognitive results, mice were tested on performance. (b) Results show high velocity by wild-type mice, (c) with similar distance swam by both IL-4Rα^-/-^ and wild-type mice. (d) IL-4Rα^-/-^ mice also showed similar decreasing latencies to platform location during the acquisition phase of the task, and (e. i) demonstrated longer latencies to platform location (e. ii) with fewer platform crossings. Data shown represents means ± SEM; n = 6 mice per group. * P < 0.05; **P < 0.01; ***P < 0.001; **** P < 0.0001 compared to wild-type mice.

To investigate the impact of IL-4Rα deficiency on cognitive behaviour, wild-type (WT) and IL-4Rα knockout (KO) mice were tested in the Morris water maze (MWM) using different paradigms, which include psychomotor task (Fig. 1B, 1C) that underscores swimming threshold velocity and distance, hippocampal-dependent spatial learning and/or reference memory tasks during the initial 4-day acquisition (Fig. 1D) and day 5 probe test (Fig. 1E), as well as reversal or complex tasks for testing behavioural flexibility (Fig. 1D) that relies on higher brain functions. We observed significant decrease in swimming velocity of IL-4Rα KO mice compared to the WT (strain: F_(1,90)_ = 21.30; *p <* 0.001; Fig. 1B; strain × time: F_(3,90)_ = 7.708; *p <* 0.001; Fig. 1B), whereas both strains of mice swam similar distances (*p > 0.05;* Fig. 1C) thus validating other behavioural tasks in the MWM that are dependent on intact locomotor function. Two-way ANOVA indicated no differences in latency to platforms in both groups of mice during acquisition (simple) and reversal (complex) learning tasks (*p > 0.05;* Fig. 1D). Interestingly, probe test performed on day 5 showed that IL-4Rα deficient mice had longer latencies to platform location (t = 4.816, *p* = 0.0085*;* Fig. 1Ei) with fewer crossings (t = 4.285, *p* = 0.0036*;* Fig. 1Eii) than the WT control mice, thus confirming that IL-4Rα is essential for hippocampal-dependent spatial reference memory task.

## 4 IL-4Rα deficiency contributes to modulated myeloid cell population numbers

**Fig. 2:**
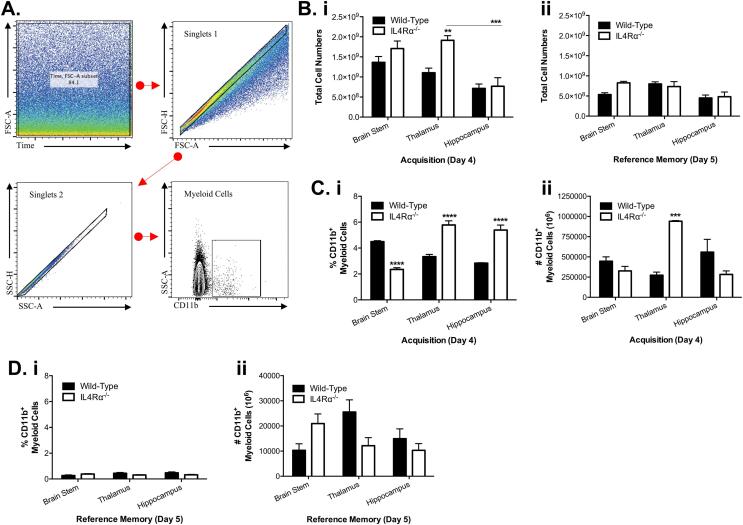
*Altered myeloid cell population in the brain during acquisition.* Single-cell suspensions from various brain areas were examined by FACS for CD11b + myeloid cells following the MWM task for wild-type and IL-4Rα^-/-^ mice. (a) A gating strategy for CD11b + myeloid cell population is shown. (b) Total cell numbers were determined from the brain stem, thalamus and hippocampus. (bi) More cells in the thalamus of IL-4Rα^-/-^ mice during acquisition, and (bii) no change in all three brain areas during reference memory. (ci) Percentage population of myeloid cells were determined, showing few CD11b + cells in the brainstem by IL-4Rα^-/-^ mice, with more cells in the thalamus and hippocampus during acquisition; (cii) specific myeloid cell count was significantly increased only in the thalamus of IL-4Rα^-/-^ mice during acquisition. (di, dii) There are no observed CD11b + cell population differences during reference memory phase of the task. Data shown represents means ± SEM; n = 6 mice per group. **P < 0.01; ***P < 0.001; **** P < 0.0001 compared to wild-type mice.

There is accumulating evidence that alternative activation of myeloid cells enhances cognitive functions (Derecki et al., 2011). Having established the behavioural effects of IL-4Rα deficiency in reference memory tasks, we decided to investigate whether this receptor regulates myeloid cell-derived immune responses in hippocampal–dependent and non-dependent substrate brain regions following MWM training. To achieve this, we employed flow cytometry to quantitatively assess CD11b + myeloid cell population in single-cell suspensions of hippocampi, thalami and brainstem of IL-4Rα KO and WT mice, owing to multi-directional communication between these brain structures. Our results show increased total cell population in the brain during acquisition more than the reference memory phase of the task (Fig. 2B). However, IL-4Rα deletion increased total cell numbers only in the thalamus on day 4 of the acquisition phase (F_(1,15)_ = 11.05; *p =* 0.0046; Fig. 2Bi) with no significant changes in all three brain areas during reference memory (day 5) (*p > 0.05;* Fig. 2Bii). Interestingly, there was interaction effect between IL and 4Rα deletion and substrate brain regions on the accumulative proportions of CD11b + myeloid cells (interaction: F_(2,15)_ = 61.32; *p <* 0.0001; Fig. 2Ci). The percentage population of CD11b + myeloid cells assessed during acquisition was significantly increased in the thalami and hippocampi of IL-4Rα KO mice, but decreased in the brainstem, when compared to the WT control mice (F_(1,15)_ = 30.39; *p <* 0.0001; Fig. 2Ci). IL-4Rα deletion also increased the numbers of viable myeloid cells only in the thalamus during MWM training (*p <* 0.0001*;* Fig. 2Cii). Curiously, very low levels of myeloid cells were detected in all the substrate brain regions on day 5 (Fig. 2Di) with no significant changes in percentage and numbers of CD11b + myeloid cells (*p > 0.05;* Fig. 2Di, 2Dii).

### 4.1 Thalamic IFN-γ decrease, and enhanced BDNF production in the absence of IL-4Rα support spatial learning

**Fig 3:**
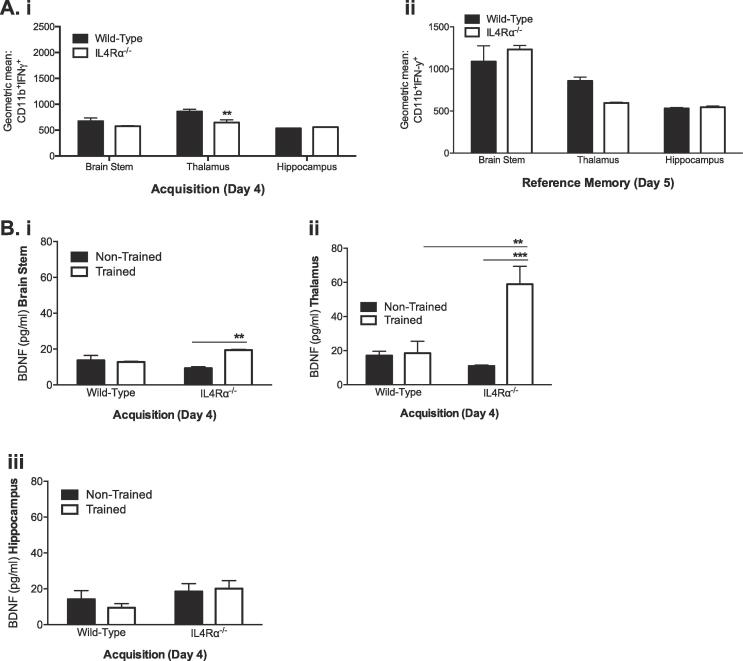
*Brain derived neurotrophic factor and Interferon gamma production influence spatial learning and reference memory.* Single-cell suspensions from the brainstem, thalamus and hippocampus were examined by FACS for geometric means of CD11b + myeloid cells positive for IFN- γ. (ai) IL-4Rα^-/-^ mice show similar levels of IFN- γ in the brainstem and hippocampus and low levels in the thalamus during acquisition; (aii) during the reference memory phase of the task, similar levels of IFN-γ were expressed in all three brain areas of IL-4Rα^-/-^ mice. BDNF was determined by means of ELISA during acquisition, showing increased BDNF by MWM-trained IL-4Rα^-/-^ mice compared to non-trained IL-4Rα^-/-^ mice in the brainstem (bi) and thalamus (bii), without any change in the hippocampus (biii). Data shown represents means ± SEM; n = 6 mice per group. * P < 0.05; **P < 0.01; ***P < 0.001; **** P < 0.0001 compared to wild-type and/or non-trained mice.

To further clarify IL-4Rα–directed myeloid cell neuromodulation of spatial learning, single-cell suspensions from the substrate brain regions were further examined for geometric means of CD11b + myeloid cells producing IFN-γ. Hippocampal CD11b + myeloid cells produced similar amount of IFN-γ during acquisition and reference memory irrespective of IL-4Rα expression or deficiency (*p > 0.05;* Fig. 3Ai, 3Aii). Whereas IL-4Rα deletion decreased IFN-γ production by thalamic myeloid cells only during the acquisition phase (strain: F_(1,17)_ = 5.824; *p =* 0.0274; Fig. 3Ai) with no significant alterations in cytokine expression in the brainstem (*p > 0.05;* Fig. 3Ai, 3Aii). It is well known that BDNF signalling is not only critical for hippocampal–dependent learning (Mizuno et al., 2000, Shaw et al., 2001, Wu et al., 2020) but also training–induced functional synaptic plasticity (O’Callaghan et al., 2007). We therefore investigated the impact of IL-4Rα deficiency on endogenous production of BDNF during acquisition by quantifying levels of this neurotrophin in the hippocampus, thalamus and brainstem of trained and non-trained mice. Deletion of IL-4Rα significantly increased BDNF production in the brainstem (training: F_(1,11)_ = 8.876; *p =* 0.0125; Fig. 3Bi) and thalamus (training: F_(1,11)_ = 18.66; *p =* 0.0012; training × strain: F_(1,11)_ = 16.60; *p =* 0.0018; Fig. 3Bii) of trained mice compared to non-trained IL-4Rα KO and WT counterparts (Fig. 3Bi,ii), whereas no differences were found in the hippocampal BDNF (*p > 0.05;* Fig. 3Biii).

### 4.2 Macrophage/microglia IFN-γ production was not altered by IL-4Rα deletion

**Fig 4:**
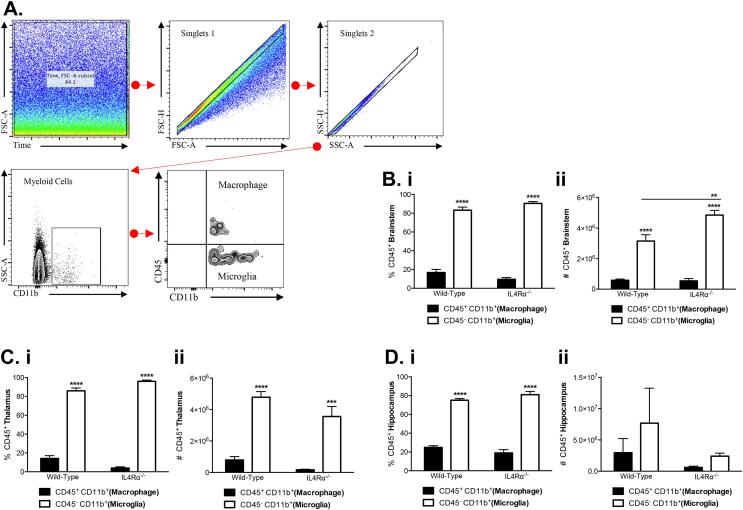
*Macrophages and microglia production during reference memory task.* Single-cell suspensions from the brain stem, thalamus and hippocampus were examined by FACS for CD45 + CD11b + macrophages and CD45-CD11b + microglia following the task of reference memory in MWM for wild-type and IL-4Rα^-/-^ mice. (a) A gating strategy for CD45 + CD11b + and CD45-CD11b + cell population is shown. To determine macrophage and microglia populations from CD11b + cells, CD45 + CD11b + and CD45-CD11b + cells were gated on, showing significantly more CD45-CD11b + cells by wild-type and IL-4Rα^-/-^ mice, compared to CD45 + CD11b + in the (b) brainstem, (c) thalamus, and (d) hippocampus. Data shown represents means ± SEM; n = 6 mice per group. * P < 0.05; **P < 0.01; ***P < 0.001; **** P < 0.0001.

**Fig. 5:**
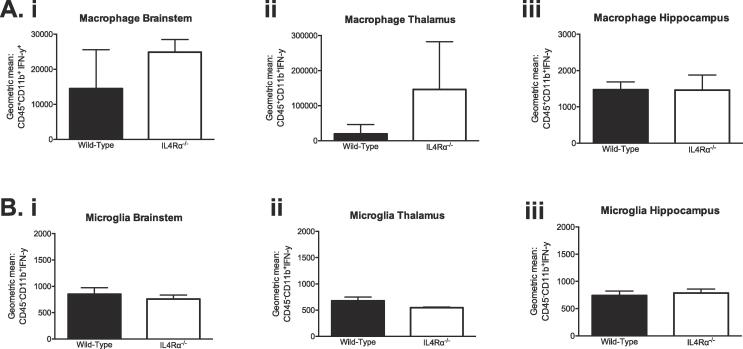
*Varying myeloid cell population following the MWM task of spatial reference memory.* Single-cell suspensions from the brain stem, thalamus and hippocampus were examined by FACS for geometric means of CD45 + CD11b + and CD45-CD11b + myeloid cells positive for IFN- γ for reference memory showing no differences between wild-type and IL-4Rα^-/-^ mice in IFN-γ production by (a) macrophages and (b) microglia in substrate brain areas. Data shown represents means ± SEM; n = 6 mice per group. * P < 0.05; **P < 0.01; ***P < 0.001; **** P < 0.0001 compared to wild-type mice.

A previous study shows that microglia/macrophage activation impaired spatial memory ability in mice (Yang et al., 2015). To further investigate biological changes associated with impaired memory tasks in IL-4Rα deficient mice, we quantified the population of brain macrophages (CD45 staining) by gating CD11b^+^ myeloid cells. Using flow cytometry, macrophages (CD45^+^CD11b^+^) were distinguished from microglia (CD45^-^CD11b^+^) in brain suspensions as previously described (Hsieh et al., 2014). As expected, the proportion and numbers of brain microglia expressed in the brainstem, thalami and hippocampi of WT and IL-4Rα KO mice were significantly higher than macrophage population (*p <* 0.001, Fig. 4B, 4C and 4D), except for lack of differences in the amount of the subset cells in the hippocampus (*p >* 0.05, Fig. 4Dii). Two-way ANOVA also showed that the amount of microglia expressed in the brainstem of IL-4Rα KO mice is greater than those expressed in WT mice (F_(1,14)_ = 8.557; *p =* 0.01; Fig. 4Bii), indicating that IL-4Rα mediates microglia response to memory tasks in this brain area. To determine the role of IL-4Rα on IFN-γ production by macrophages and/or microglia, we evaluated geometric means of CD45^+^CD11b^+^ and CD45^-^CD11b^+^ myeloid cells positive for IFN-γ. Surprisingly, there were no differences in IFN-γ production in all three brain areas of IL-4Rα KO and WT mice (*p >* 0.05, Fig. 5A, 5B).

## 5 Discussion

In this study, we demonstrate that inhibited cytokine signalling through IL-4Rα chain disrupts psychomotor performance and reference memory in mice, while sparing behavioural phenotype associated with spatial learning. These IL-4Rα–mediated behavioural effects are generally characterized by accumulative proportions and activation of CD11b + myeloid cells and decreased expression of IFN-γ, mostly in the thalamus during acquisition, and favoured by IL-4Rα deletion. These changes evoked simultaneous increases in endogenous BDNF production in the thalamus and brainstem of trained mice. We have also identified that IFN-γ behavioural effects are not only regionally selective but also its expression appears limited to non-gated CD11b + myeloid cells, despite spiked microglia response influenced by IL-4Rα deficiency during the reference memory tasks.

Although previous studies including reports from our laboratory have indicated the beneficial impacts of IL-4 and/or IL-13 on learning and memory functions (Brombacher et al., 2017, Derecki et al., 2010), the precise role of their heterodimeric IL-4Rα in cognitive–related tasks is yet to be validated. Combining loss-of-function approach with behavioural paradigms, we show for the first time that absence of IL-4Rα selectively alters swim velocity and performance on spatial reference memory tasks that are highly dependent on intact hippocampal functioning. Since learning remains unaffected by the receptor inhibition, our findings confirmed previous reports that support the involvement of other higher brain areas, including thalamus and brainstem, mediating learning and/or cognitive processes (Lopez et al., 2009, Loureiro et al., 2012, Yagi et al., 1998).

Interestingly, our study partly supported the findings by Derecki et al (2011) that alternative activation of myeloid cells enhance cognitive functions in immune compromised mice (Derecki et al., 2011), as we established that IL-4Rα deletion differentially regulates myeloid cell activity in the three brain areas examined to influence acquisition of learned tasks, but not retention. Low levels of brain CD11b + myeloid cells during spatial memory task may also be responsible for the observed impaired memory irrespective of the receptor deficiency. Understanding the brain microenvironments and susceptibility to immune challenge, like swimming stress, likely impacts on myeloid cell contribution to spatial learning. In a recent study, region-specific alterations in myeloid cell composition was detected in distinct brain regions of TNFtg (TNFα transgenic mouse model of rheumatoid arthritis) mice with rapid activation in the cortex, striatum and thalamus, but very limited alterations in the hippocampus and cerebellum (Süß et al., 2020). In this study, we presume that topographic clustering of myeloid cells in the thalamus of IL-4Rα KO mice is based on sensitivity and complement response to compromised immunity and swim stress.

Several recent studies have shown that the proinflammatory cytokine IFN-γ act as a negative regulator of hippocampal functions (Flood et al., 2019, Monteiro et al., 2016). For example, mice lacking IFN-γ have been shown to exhibit enhanced cognitive performance, increased hippocampal plasticity and pre-synaptic neurotransmitter release Litteljohn et al., 2014, Monteiro et al., 2016). Also, reduced gamma oscillations in the hippocampal CA3 regions have been linked to chronic IFN-γ treatment (Ta et al., 2019), while Maher et al (2006) had previously shown that intracerebroventricular injection of IFN-γ impaired induction of long-term potentiation in the hippocampal brain slices of rats (Maher et al., 2006). To date, no plausible molecular mechanism has yet been offered to account for the effects of IL-4Rα–directed expression of IFN-γ on myeloid cells linked to cognitive functions. We show in this paper that mice deficient of IL-4Rα had decreased expression of myeloid cell-derived IFN-γ in the thalamus only during the acquisition phase without significant changes in other brain areas. It is not surprising that low levels of IFN-γ were generally expressed in all three substrate brain regions since a previous study reported likewise (Rady et al., 1995). Notably, decreased thalamic concentration of IFN-γ suggests neuromodulation of this proinflammatory molecule by IL-4Rα which may further account for the unabated spatial learning. It remains unclear why IFN-γ expression in the hippocampus is unaffected by memory task, nonetheless our results are in line with a previous study that both IFN-γ KO and WT mice were able to learn successfully the spatial reference memory task, except that deletion of IFN-γ is expected to enhance cognitive performance (Monteiro et al., 2016), most notably improved or unimpaired memory. One possible suggestion for the memory-related discrepancy is that we suspect that deletion of IL-4Rα partially shut down the activity of IFN-γ precursory molecules in the myeloid cells and/or functional receptors in the brain. Importantly, it is expected that these changes may influence further the pleiotropic nature of IFN-γ molecule, as previously posited that IFN-γ KO mice exhibited memory disturbances at basal states whereas memory performance facilitated by chronic stress was accompanied by altered noradrenergic and serotonergic activity in the hippocampus (Litteljohn et al., 2014).

Spatial learning and performance in memory tasks have been linked to hippocampal BDNF content (Petzold et al., 2015, Wu et al., 2020). In the current study, we demonstrate an important role of IL-4Rα-mediated BDNF activity in the brain and show that IL-4Rα deletion favours training-induced BDNF production in the brainstem and thalamus which correlates to the uninterrupted spatial learning during acquisition phase. Since both mouse strains including IL-4Rα KO were able to learn the assigned spatial tasks, we therefore expect positive correlation between hippocampal BDNF levels and the probe tasks. Surprisingly, insignificant change in hippocampal BDNF levels were observed which is believed to further account for the impaired reference memory in our mice. A recent study has shown that inhibition of microglial cell activation leads to improved spatial memory (Wadhwa et al., 2017). Using cell gating technique, we show that an increase in microglia population in all three brain areas in the absence of IL-4Rα correlates to poor reference memory, suggesting sensitization and/or activated response by these brain resident cells. For many years, this primed process has been identified in aged brain, neurodegenerative or neuroinflammatory conditions leading to memory impairments (Barrientos et al., 2010, Hou et al., 2014). It is therefore believed that activated microglia response secondary to the immune challenge eroded the capacity of hippocampal cells to produce sufficient BDNF that is required for memory-related plasticity (Patterson, 2015). Since it has been reported that IFN-γ regulates microglial functions differently from that seen in spectrum of monocytically derived macrophages (Colton et al., 1992), we expect that microglia-derived IFN-γ would further complement microglial function but its quiescence suggests no complementary biological effects in this model.

In conclusion, we show that IL-4Rα deficient mice can learn MWM spatial tasks like their WT counterparts by a mechanism that involves modulation of brain CD11b + myeloid cells, primed microglial response and decreased IFN-γ expression. While uninterrupted spatial learning was further supported by training-induced BDNF production in the brainstem and thalamus, we observed that successful retention of memory-related probe tasks depends strongly on hippocampal BDNF with only little influence by other factors.

## Declaration of Competing Interest

The authors declare that they have no known competing financial interests or personal relationships that could have appeared to influence the work reported in this paper.
